# Design, physicochemical characterisation, and *in vitro* cytotoxicity of cisplatin-loaded PEGylated chitosan injectable nano / sub-micron crystals

**DOI:** 10.1016/j.jsps.2023.04.005

**Published:** 2023-04-13

**Authors:** Muhammad H. Sultan, Sivakumar S. Moni, Saad S. Alqahtani, Mohammed Ali Bakkari, Abdulrahman Alshammari, Yosif Almoshari, Saeed Alshahrani, Osama A. Madkhali, Syam Mohan

**Affiliations:** a Department of Pharmaceutics, College of Pharmacy, Jazan University, Jazan, Saudi Arabia; b Department of Clinical Pharmacy, College of Pharmacy, Jazan University, Jazan, Saudi Arabia; c Pharmacy Practice Research Unit, College of Pharmacy, Jazan University, Jazan, Saudi Arabia; d Department of Pharmacology and Toxicology, College of Pharmacy, King Saud University, Riyadh, Saudi Arabia; e Department of Pharmacology and Toxicology, College of Pharmacy, Jazan University, Jazan, Saudi Arabia; f Substance Abuse and Toxicology Research Centre, Jazan University, Jazan, Saudi Arabia

**Keywords:** Cancer, Cisplatin, Nanoparticles, Pegylated Chitosan, Zetapotential, Polydispersity Index, Size, Cytotoxicity

## Abstract

The study aimed to develop cisplatin-loaded PEGylated chitosan nanoparticles. The optimal batch of cisplatin-loaded PEGylated chitosan nanoparticles had a + 49.9 mV zeta potential, PDI of 0.347, and % PDI of 58.9. Nanoparticle zeta size was 741.4 z. d.nm, the size in diameter was 866.7 ± 470.5 nm, and nanoparticle conductivity in colloidal solution was 0.739 mS/cm. Differential scanning calorimetry (DSC) revealed that cisplatin-loaded PEGylated chitosan nanoparticles had sharp endothermic peaks at temperatures at 168.6 °C. The thermogravimetric analysis (TGA) showed the weight loss of cisplatin-loaded PEGylated chitosan nanoparticles, which was observed as 95% at 262.76 °C. XRD investigation on cisplatin-loaded PEGylated chitosan nanoparticles exhibited distinct peaks at 2θ as 9.7°, 20.4°, 22.1°, 25.3°, 36.1°, 38.1°, 39.5°, 44.3°, and 64.5°, confirming crystalline structure. The ^1^H NMR analysis showed the fingerprint region of cisplatin-loaded PEGylated chitosan nanoparticles as 0.85, 1.73, and 1.00 ppm in the proton dimension and de-shielded proton peaks appeared at 3.57, 3.58, 3.58, 3.59, 3.65, 3.67, 3,67, 3,67, 3.70, 3.71, 3.77, 3.78 and 4.71 ppm. The ^13^C NMR spectrum showed specified peaks at 63.18, 69.20, and 70.77 ppm. The FT-IR spectra of cisplatin loaded PEGylated nanoparticles show the existence of many fingerprint regions at 3186.52, 2931.68, 1453.19, 1333.98, 1253.71, 1085.19, 1019.60, 969.98, 929.53, 888.80, 706.13, and 623.67 cm^−1^. The drug release kinetics of cisplatin loaded PEGylated chitosan nanoparticles showed zero order kinetics with 48% of drug release linearity fashion which has R^2^ value of 0.9778. Studies on the MCF-7 ATCC human breast cancer cell line *in vitro* revealed that the IC50 value 82.08 µg /mL. Injectable nanoparticles had good physicochemical and cytotoxic properties. This method is novel since the application of the PEGylation processes leads to an increased solubility of chitosan nanoparticles at near neutral pH.

## 1 Introduction

Multiple drug resistance is a major hurdle during the treatment of cancer and has become a health problem worldwide (Behzad et al., 2017, Abouelhag et al., 2017). The utilization of chemotherapy with cytotoxic drugs to treat cancer not only destroys cancer cells but also nearby noncancerous cells (Hu and Zhang, 2009). The development of resistance in a cancer cell can occur through a variety of cellular and molecular pathways, which results in a partial response to treatment. Resistance may be brought on by the ineffective or inadequate delivery of cytotoxic medications at the location of the tumor (Lizbeth et al., 2017, Hiba and Katherine, 2013). Molecularly, genetic mutation is the main mechanism of the drug resistance developed by cancer cells and leads to changes in cellular signalling pathways and the overexpression of drug efflux pumps. The previous therapeutic era focused on multi-drug therapy and combination drug therapy with different target mechanisms that exert better efficacy than mono-drug therapy (Che-Ming and Liangfang, 2009). However, combination therapy also leads to treatment failure by the development of drug resistance. The therapeutic index of a drug molecule can be enhanced by delivery at the specific target cell. This strategy will solve many problems related to drug resistance and drug-induced toxicity. Moreover, anticancer drugs must overcome pharmacokinetic (PK) resistance, which affects conventional cytotoxic drugs and targeted therapies (Wenbo et al., 2018). Nanoparticles have the potential to improve the therapeutic compatibility of targeted cancer treatments due to their ability to enhance the pharmacokinetic (PK) properties of drug delivery at the site of cancer cells. This is since their endogenous transport system through the cell membrane is made easier due to the nanoscale size of the particles (Moni et al., 2020, Au et al., 2016, Che-Ming and Liangfang, 2009).

Chitosan is a polycationic copolymer of glucosamine and N-acetylglucosamine. It is obtained by the deacetylation of chitin, an abundantly available natural polymer from crustaceans. Chitosan is a weak base that is insoluble in water and organic solvents, soluble in dilute acidic solutions (pH < 6.5) and forms a precipitate with an alkaline solution. Owing to the biodegradability, biocompatibility, nontoxicity, high charge density, mucoadhesive properties of chitosan, and it has potential applications in pharmaceuticals (Zaigang et al., 2023, Jiashe et al., 2022, Zaigang et al., 2022, Dash et al., 2011, Amidi et al., 2010, Paul, 2010, Claus-Michael et al., 1992). Generally, chitosan is soluble at an acidic pH owing to protonation (Singh and Ray, 2000, Rinaudo, 2008). The deacetylation process influences chitosan solubility as 85% of the deacetylated polymer is soluble at an acidic pH (6.5). A high degree of deacetylation leads to high solubility in water (Yong-Woo et al., 2000). Cisplatin is a chemotherapeutic drug used to treat several human cancers, including breast cancers, non-Hodgkin lymphoma, bladder, head and neck, lung, ovarian, and testicular cancers (Shaloam and Paul, 2014). Although cisplatin is successful in the treatment of various cancers, severe toxicity and drug resistance have been reported (Abouelhag et al., 2017, Bradley et al., 2014, Galluzzi et al., 2012, Yousef et al., 2009). Nanoparticles can deliver the drug in a naïve form that augments the maximal plasma concentration of the drug, reduces the dosage, and overcomes toxicity (Jayanta et al., 2018). However, the safety and tolerability of injectable chitosan nanoparticles are key considerations. Therefore, PEGylation is used to minimize the limitations of chitosan, such as precipitation at near neutral pH, and to increase solubility and tolerability. The PEGylated chitosan nanoparticles were developed by an ionic gelation technique with modification. This was a one-step process in which formaldehyde was used as a cross linker between chitosan and polyethylene glycol (PEG). The concentration of cross linker formaldehyde was kept low to avoid cellular toxicity. This study was focused to determine the physicochemical characterisation and cytotoxicity efficacy of the formulated cisplatin-loaded PEGylated chitosan nanoparticles.

## 2 Materials and methods

### 2.1 Materials

Lower molecular weight chitosan with a 75% degree of deacetylation, 200–300 cps viscosity grade, was purchased from Merck, Darmstadt, Germany. Cisplatin, PEG 400, and formaldehyde were purchased from Sigma Aldrich, St. Louis, MO, USA. All other chemicals and solvents were supplied by Somatco, Jeddah, Saudi Arabia.

### 2.2 Methods

#### 2.2.1 Formulation of nanoparticles

Cisplatin-loaded PEGylated chitosan nanoparticles were prepared using an ionic gelation technique. Initially, 1% w/v chitosan polymer was prepared in 1% v/v glacial acetic acid. The chitosan gel was kept overnight to stabilise. Then, a 1% w/v cisplatin solution was prepared in phosphate-buffered saline at pH 7.4. PEG (50% w/v) was used as the grafting polymer for the PEGylation of the chitosan polymer. Formalin solution (0.25% v/v) was used as the cross-linker to the chitosan based on ionic attraction. Formaldehyde also reacted with PEG because PEG has a free OH group. Initially, the chitosan gel was transferred to a separate clean glass beaker and PEGylation was performed by the dropwise addition of 50% w/v PEG. The mixture was homogenised (3,000 rpm / 30 min) and stirred at 80 °C. During PEGylation, 1% w/v cisplatin was added, and the homogenisation process continued for 15 min. Then, a 0.25% v/v formalin solution was added as the cross-linker and acted as a catalyst for the reaction between chitosan polymer and PEG. The reaction mixture was homogenised at the specified temperature for 60 min after the addition of formaldehyde. During nanoparticle formulation, the mixture was sonicated for 5 min at 80% amplification three times at predetermined time intervals. After formulation, the mixture was allowed to cool. Then, it was filtered through polyvinylidene fluoride membrane filters with a 0.45 µm pore size. The filtrate was further filtered using a syringe filter with a 0.22 µm pore size. The filtrate was collected in amber coloured vials, closed, and stored in a refrigerator at 2–8 °C for further analysis. The formulation was standardised based on extensive preformulation studies, which were carried out by varying the concentrations of chitosan polymer, PEG, and formaldehyde.

#### 2.2.2 Measurement of pH

The pH is the measure of acidity or alkalinity of liquid or colloidal samples. The pH of nanoparticle reaction mixture was measured by Oakton pH 700, a benchtop meter (Oakton Instruments, Vernon Hills, IL, USA).

#### 2.2.3 Lyophilization process

The lyophilization was performed by using Millrock BT85 desktop freeze dryer (Millrock Technology, USA). In a glass flask, a reaction mixture was prepared by mixing PEGylated nanoparticles and 5% w/v mannitol in 1:1 vol ratio. The mixture was placed in a deep freezer at −80 °C for 24 h for freeze drying. The PEGylated nanoparticles were subsequently placed in vacuum-controlled lyophilizing tubes. The vacuum was maintained at 3000 Pa and the temperature was maintained at −84 °C. The lyophilized PEGylated nanoparticles were eluted from the glass flask after 24 h, then pooled, and stored at 4 °C for further analysis.

#### 2.2.4 Dynamic light scattering (DLS) analysis

The nanoparticles were physically characterized by measuring their zeta potential (ZP) in millivolts (mV), their conductivity in milli siemens per centimeter (mS/cm), their pH, their size in nanometres (d.nm and z. d.nm), and their polydispersity index (PDI). Briefly, 10% w/v of lyophilized nanoparticles were prepared in Milli Q water, placed in capillary cells, and physical characterization of nanoparticles was performed by using Zetasizer Nano NS, Malvern Instruments, Malvern, UK (Sultan et al., 2022).

#### 2.2.5 Determination of morphological features

The morphological features and particle size of the formulated nanoparticles were studied using a high-resolution scanning electron microscopy study. Using a JEOL JSM 6360 (JEOL USA, Inc, Japan) scanning electron microscopy (SEM) with high resolution, the morphological properties of lyophilized PEGylated nanoparticles were analysed. The morphological characters were investigated under reduced pressure by spreading lyophilized PEGylated nanoparticles on metal stubs and coated with gold–palladium to a thickness of between 200 and 300 Å (Sultan et al., 2022).

#### 2.2.6 Differential scanning calorimetry (DSC) analysis

Lyophilized PEGylated nanoparticles were analyzed by means of the DSC method to ascertain the enthalpy changes that occurred because of changes in the physical and chemical properties of the samples. The Shimadzu DSC 60 (Japan) was utilized to carry out the DSC analysis on the PEGylated chitosan nanoparticles. In an un hermetically sealed aluminium pan, nanoparticle powder was placed, and the temperature was increased from 30 to 250 °C at a rate of 10 °C per minute while maintaining a 10 mL/min airflow min^−1^ (Madkhali et al., 2021).

#### 2.2.7 Thermogravimetric analysis (TGA)

A thermogravimetric analyzer (TGA 8000, Perkin Elmer, Waltham, MA, USA) was utilized to determine the thermal stability of the lyophilized nanoparticles. After placing the lyophilized powder PEGylated nanoparticles (10 mg) on an aluminium pan, the test was carried out at a heating rate of 10 °C per minute within a temperature range of 50 °C to 300 °C (Madkhali et al., 2021).

#### 2.2.8 X-ray diffraction (XRD) analysis

X-ray diffraction (XRD) was utilized so that the crystalline structures of lyophilized PEGylated nanoparticle powder samples could be investigated. The lyophilized nanoparticle powder was subjected to XRD examination using Unisantis XMD 300 X-ray powder diffractometer (Unisantis Europe GmbH, Germany). The XRD diffractograms at *2θ* in the range of 2-50° at a voltage of 45 kV and a current of 0.8 mA were produced using Cu K α radiation from the incoming beam (λ = 1.5418 Å). A scanning range of *2θ/θ* was selected and scanning speed of 10 min^− 1^ was employed.

#### 2.2.9 Fourier transformed infrared (FT-IR) spectroscopy

FT-IR spectroscopy is sensitive to the chemical surface of nanoparticles and helps in the identification of the functional groups and bonds before and after PEGylation of chitosan. The functional groups of the samples were analyzed by using Bruker FT-IR spectroscopy, USA. KBR pellet technique was followed, and the spectra of the pellet sample were obtained at 400–4000 cm^−1^ with a resolution of 4 cm^−1^.

#### 2.2.10 Loading and encapsulation efficiency study

The release of cisplatin from PEGylated chitosan nanoparticles were determined by establishing a standard curve as reported very recently (Sultan et al., 2022). Entrapped cisplatin was released after being suspended for 30 min in 10 mL of 0.1 N HCl solution containing 5 g of lyophilized PEGylated chitosan nanoparticles. The reaction mixture was centrifuged at 2,000 RPM, and the supernatant was collected and stored at 2 °C. The cisplatin concentration was estimated from the cisplatin calibration standard curve. Then, using the following equations, the encapsulation efficiency (EE) and drug loading (DL) were calculated:

$$\mathrm{EE}\left(\%\right)=\frac{\left(Amount\;of\;drug\;incorporated)-(amount\;of\;free\;drug\;after\;extraction\right)}{\left(Amount\;of\;drug\;incorporated\right)}\times 100$$

$$\mathrm{DL}\left(\%\right)=\frac{\mathrm{Total}\;\mathrm{amount}\;\mathrm{of}\;\mathrm{drug}\;\mathrm{extract}\mathrm{ed}\;\mathrm{from}\;\mathrm{the}\;\mathrm{nanoparticles}}{\mathrm{Total}\;\mathrm{weight}\;\mathrm{of}\;\mathrm{drug}\;\mathrm{loaded}\;\mathrm{nanoparticles}}\times 100$$

### 2.3 *In vitro* release study

This process involved placing 500 mg of lyophilized PEGylated chitosan nanoparticles in a dialysis bag and immersing them separately in 50 mL of phosphate buffer saline (pH 7.4 / 37 °C) with a magnetic bead stirring at 1000 rpm for 6 h. The initial sampling was done after 30 min to evaluate the burst release phase. After that, 3 mL of the medium was taken out of the tube each 1 h. The samples were analyzed using UV/visible spectroscopy at 265 nm, and the release pattern was determined by extrapolating the optical density against cisplatin concentration.

### 2.4 *In vitro* cytotoxicity study

The experiment was carried out in an accordance with the procedure that had been reported by Sultan et al. 2022. During this procedure, human breast cancer cells from the MCF-7 ATCC strain were cultivated and maintained in RPMI-1640 at a pH of 7.4 using a sodium bicarbonate buffer system with a concentration of 2.0 g/L. Cells were cultivated individually in a CO_2_ incubator (Heraeus, Germany) at 37 °C, 90% relative humidity, and 5% carbon dioxide. 10% foetal bovine serum (FBS), 100 u/mL penicillin, and µg/mL streptomycin were added to the medium as supplements. The PEGylated chitosan nanoparticles, dissolved in DMSO, were administered to the cells in separate treatments of 100 µL each at different concentrations (maximum dose = 100 µg/mL). Cells were seeded in 96-well microtiter plates at a density of 1 × 10^6^ cells/mL. (Treated and control). The test samples were plated in triplicate (n = 3), and then allowed to grow for forty-eight hours in an incubator containing carbon dioxide. After incubation, 20 µL of MTT with a concentration of 5 mg/mL was added to each well of the plate, and the plates were then incubated in the dark for another 4 h before the media was discarded., the test samples were plated in triplicate (n = 3), and they were let to grow for 48 h. The test samples were plated in triplicate (n = 3), and then allowed to grow for forty-eight hours in CO_2_ incubator. After incubation, 20 µL of MTT with a concentration of 5 mg/mL was added to each well of the plate, and the plates were then incubated in the dark for another 4 h before the media was discarded. After this, 100 µL of DMSO was used to dissolve the formazan crystals that had formed in each well, and the absorbance of each well was measured at 490 nm using a microtiter plate reader (Biotek ELISA reader, ELX 800, USA). After considering the appropriate controls, the percentage of cellular viability could then be calculated. The experiment was performed in triplicate, and the % inhibition of cell propagation was calculated using the formula:

$$\mathrm{Growth}\;\mathrm{inhibition}\;\left(\%\right)=\frac{\left(OD\;control-OD\;treated\right)}{\mathrm{OD}\;\mathrm{control}}x100$$

## 3 Results and discussion

### 3.1 Physical and morphological characterization

**Fig. 1:**
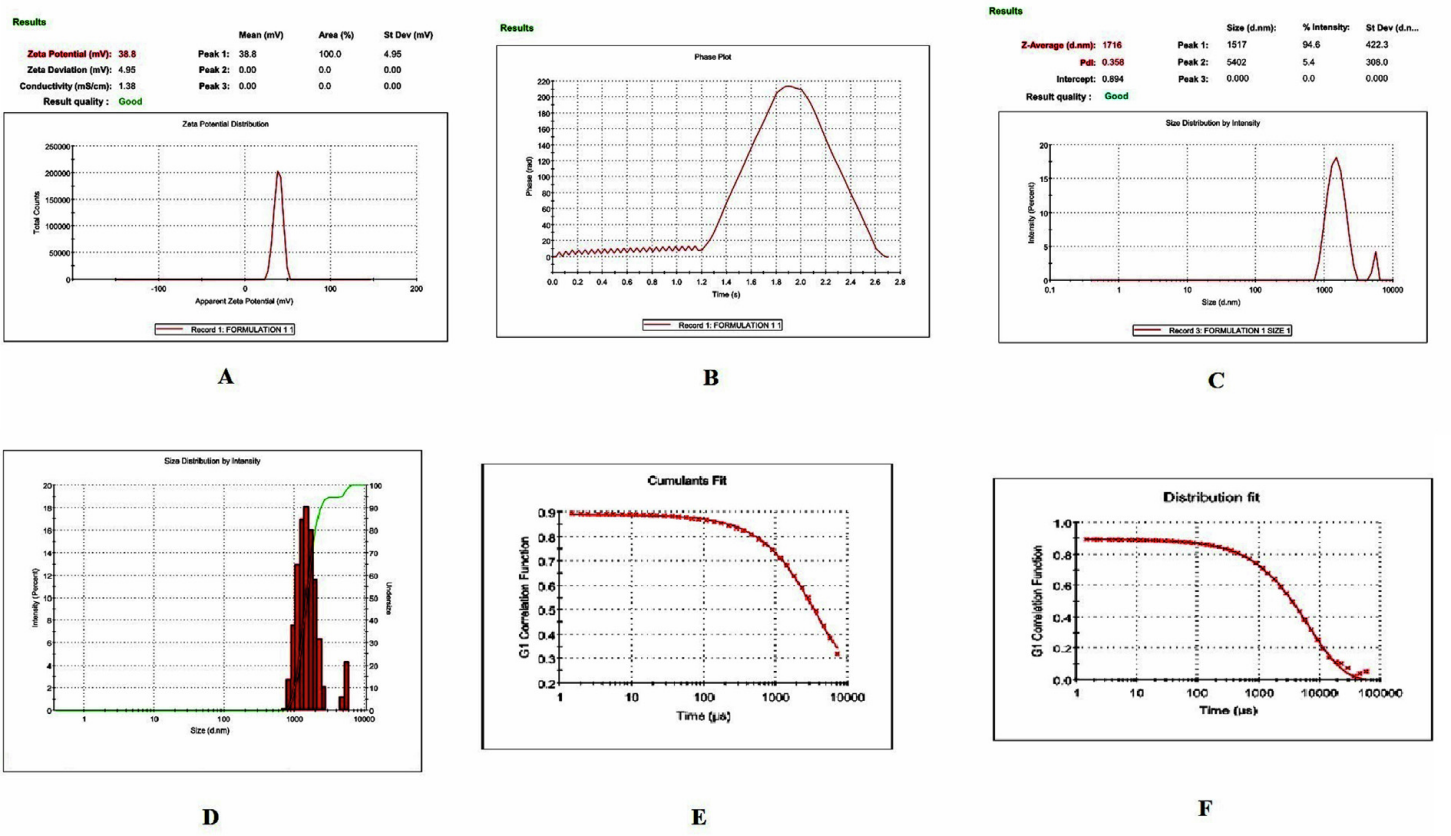
**Physical characterization of cisplatin-loaded PEGylated chitosan nanoparticles of batch 1** (A) Zetapotential analysis (B) Phase plot of particulate colloidal system (C) Particle Size distribution analysis through intensity (D) Particle Size distribution analysis (E) Cumulative fit of particulate colloidal system (F) Particle distribution fit of particulate colloidal system.

**Fig. 2:**
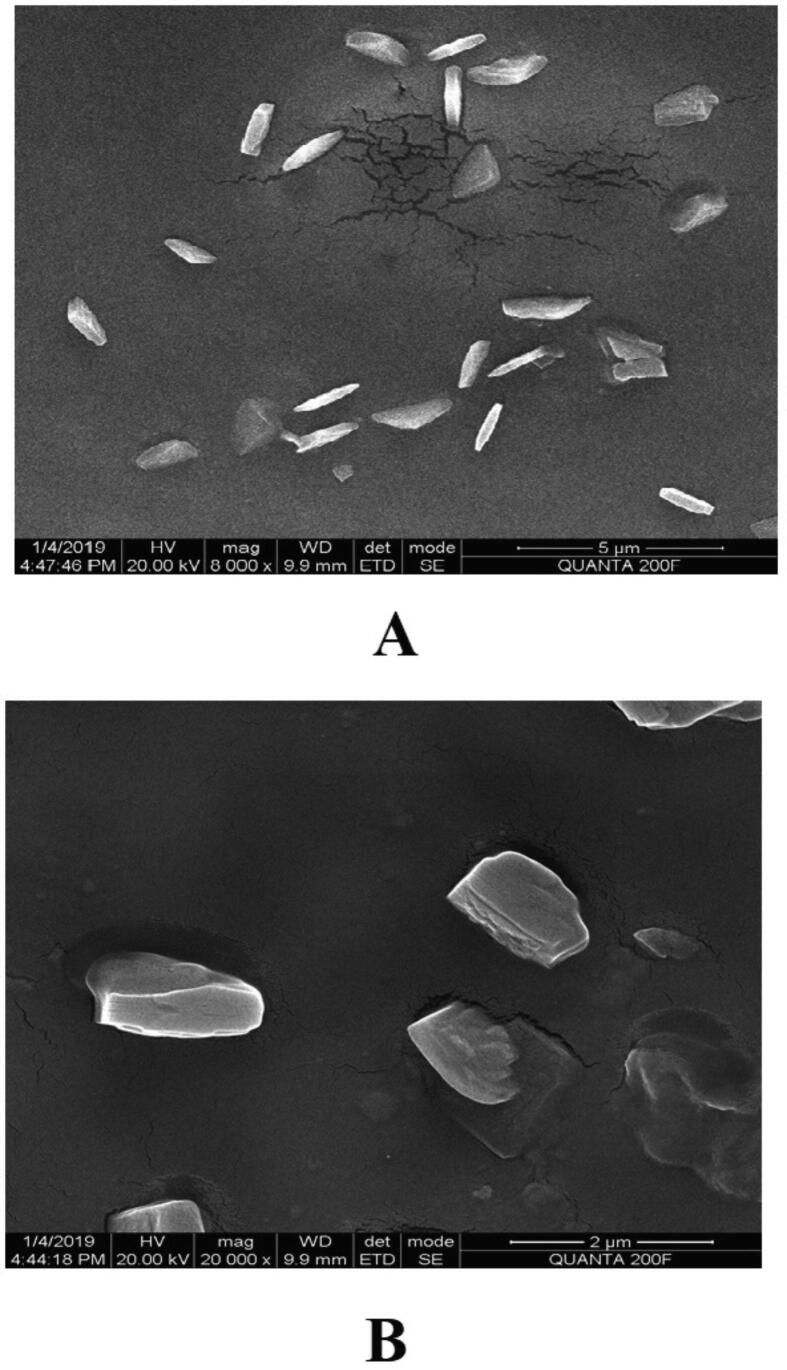
**Scanning electron micrograph study of batch 1** (A) Cisplatin-loaded PEGylated chitosan nanoparticles of batch 1 under 8,000 × magnification (B) Cisplatin-loaded PEGylated chitosan nanoparticles of batch 1 under 20,000 × magnification.

**Fig. 3:**
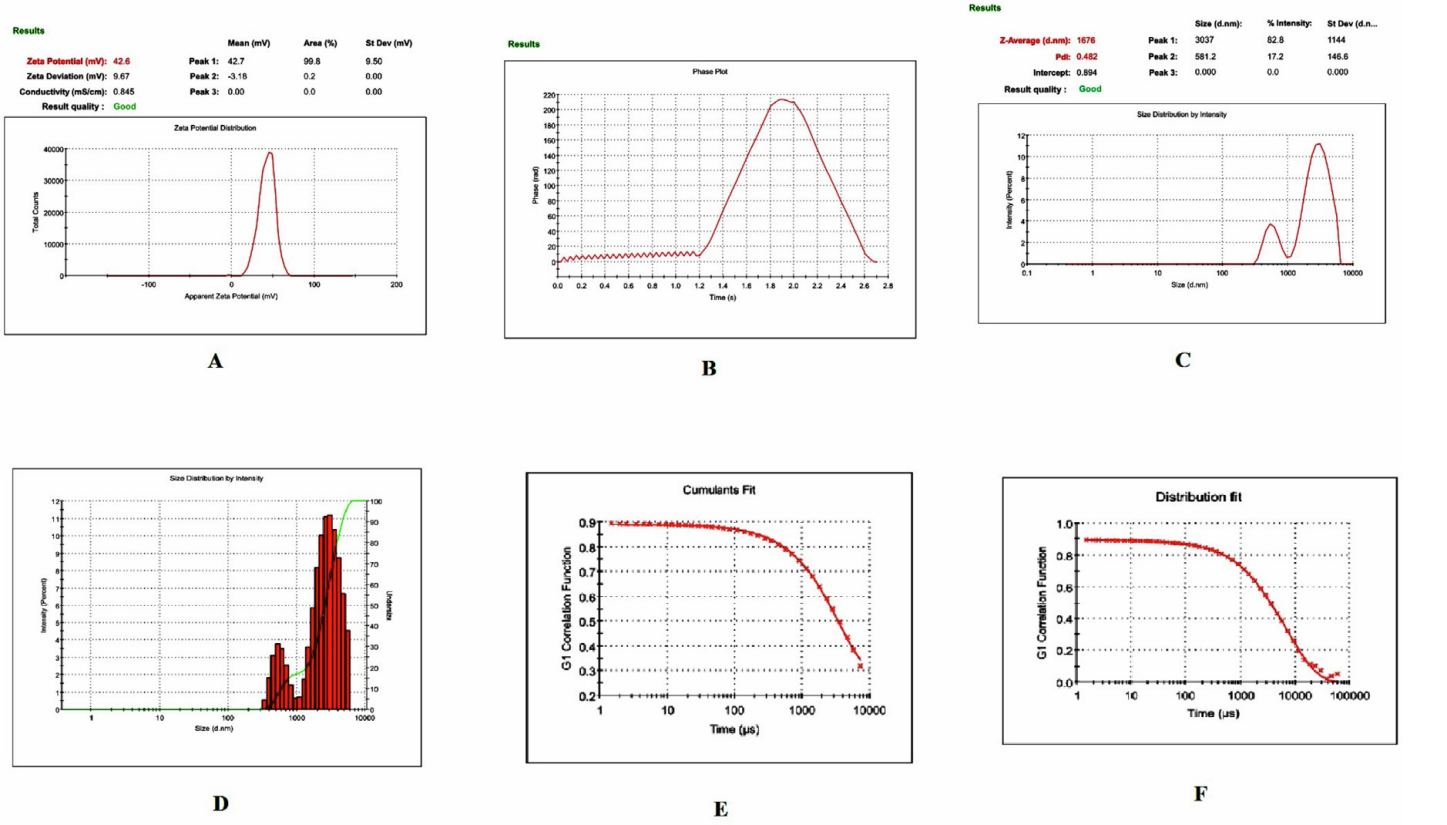
**Physical characterization of cisplatin-loaded PEGylated chitosan nanoparticles of batch 2** (A) Zetapotential analysis (B) Phase plot of particulate colloidal system (C) Particle Size distribution analysis through intensity (D) Particle Size distribution analysis (E) Cumulative fit of particulate colloidal system (F) Particle distribution fit of particulate colloidal system.

**Fig. 4:**
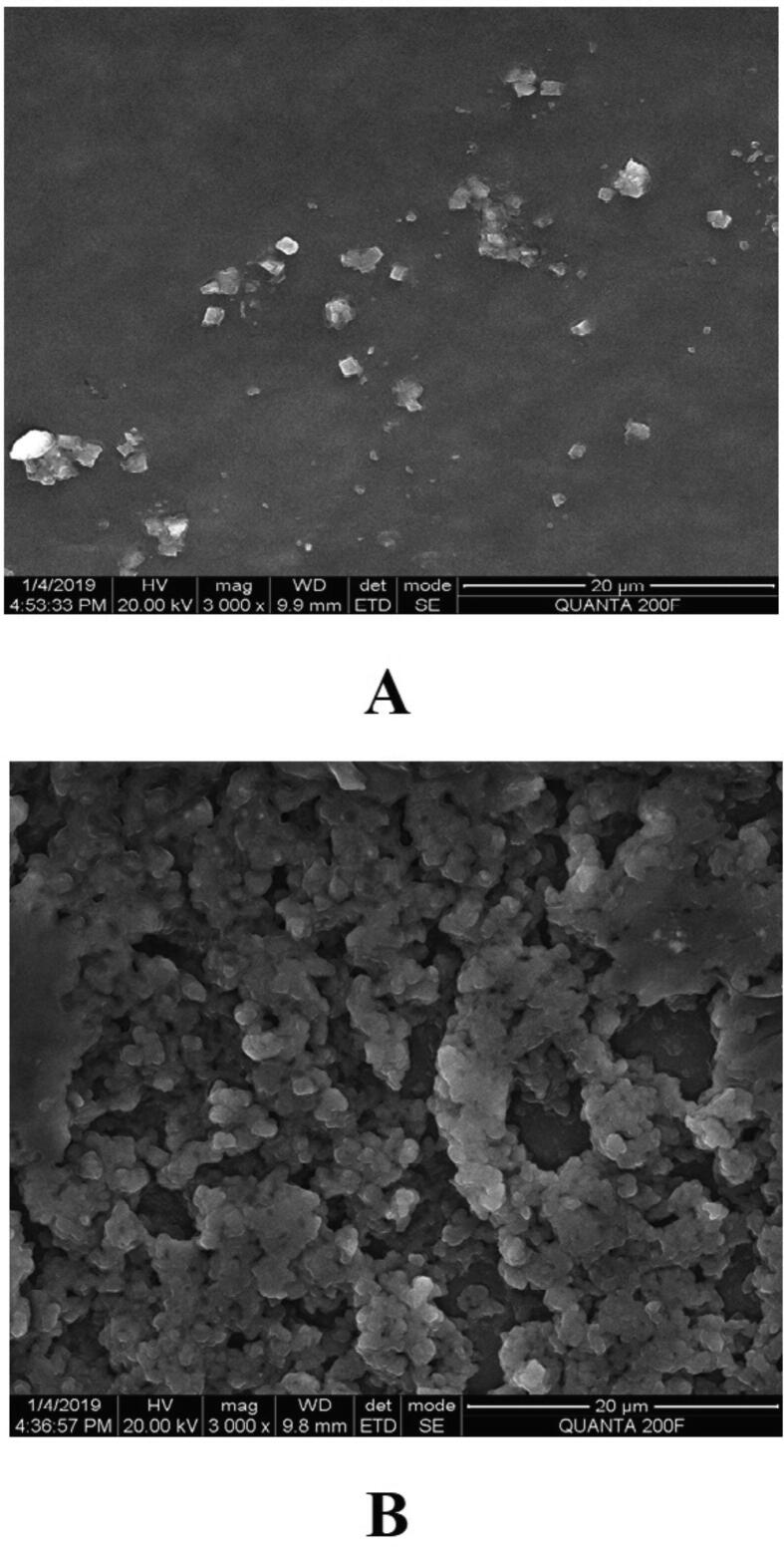
**Scanning electron micrograph study of batch 2 (**A) The scanning electron micrograph of cisplatin-loaded PEGylated chitosan nanoparticles of batch 2 under 3000 × magnification (B) The scanning electron micrograph of cisplatin-loaded PEGylated chitosan nanoparticles of batch 1 under 3,000 × magnification.

Prior to formulation, extensive preformulation studies were performed by varying chitosan polymer, PEG, and drug concentration. However, in all formulations, the formaldehyde concentration maintained at 0.25% v/v. Particle size and PDI varied with different chitosan: PEG: drug ratios. The preformulation studies produced three formulations with good physical properties (Table 1). Batch 1 nanoparticles showed a good ZP. The ZP of these nanoparticles was + 38.8 ± 4.95 mV with a unique peak. The conductivity was 1.38 mS/cm; thus, the particles had good electrostatic conduction and were electrostatically active (Fig. 1A). The nanoparticle mixture was uniformly formed and highly homogenous in a single phase in the colloidal system, with a PDI value of 0.35 (Fig. 1B). The batch 1 particles were exhibiting 59.8 % PDI with 94%intensity in a colloidal injectable form. The zeta average particle was observed as 1716 z.d.nm. The particles were large and greater than the nano range. The figure shows that 94.6% of nanoparticles were successfully formed, whereas the remaining 5.4%, with a short peak, were showing the larger particles might be due to aggregation in a colloidal injectable formulation (Fig. 1C). It is obvious that most of the particle size was showing above 1000 µm in diameter in nano meter (Fig. 1D). The cumulative fit analysis revealed a linearity of greater than 99.9%, while the size distribution fit was depicted at approximately 95% in a colloidal dispersion system (Fig. 1E & F). The Y-intercept value was determined as 0.894, which showed that this was a good injectable colloidal system. Batch 1 formulated nanoparticles showed a crystal morphology: an irregular shape with rough surfaces at 8000 × magnification (Fig. 2A). Fig. 2B demonstrating the crystal morphology with irregular shape magnified under 20,000 × in scanning electron microscopy. Batch 2 particles exhibited a good ZP of + 42.6 ± 9.67 mV (Fig. 3A). The size distribution showed a unique pattern and particles were large. The phase distribution was highly homogenous in the colloidal system (Fig. 3B), which was reflected by the PDI value of 0.482 (Fig. 3C). The size distribution of particles was showed the majority of particles exhibited above 2000 d.nm (Fig. 3D). However, the cumulative fit and size distribution fit were achieved as like batch 1 (Fig. 3E & F). The Y-intercept value was determined as 0.894, which makes it good injectable formulation. The particles had a distinctive electric potential, with a conductivity of 0.845 mS/cm which was lesser than batch 1. The particles were discrete crystals at 3000 × magnification (Fig. 4A). However, some particles were observed as clumped crystals (Fig. 4B), which supports the nanoparticles showed incomplete PEGylation.

**Table 1:** Physical characterization of cisplatin-loaded PEGylated chitosan nanoparticles.

S.no	Batches	Chitosan: PEG	Chitosan: Drug	pH	PEG: Drug	Particle characterization							
						**Zeta Potential (mV)**	**Conductivity (mS/cm)**	**PDI**	**% PDI**	**% Int**	**Particle size (nm)**	**Particle size, Zeta average (z d.nm)**	**% Mass d.nm**
1	Batch 1	5:1	10:1	6.5	2:1	38.8 ± 4.95	1.38	0.35	59.8	94.6	1517 ± 422.3	1716	62.19
2	Batch 2	10: 1	5: 2	6.5	1:10	42.6 ± 9.67	0.845	0.482	69.5	89.2	3037 ± 1144	1676	92.19
3	Batch 3	20: 1	5:2	6.5	1:10	49.9 ± 5.17	0.739	0.347	58.9	95.5	866.7 ± 470.5	741.4	51.14

**Fig. 5:**
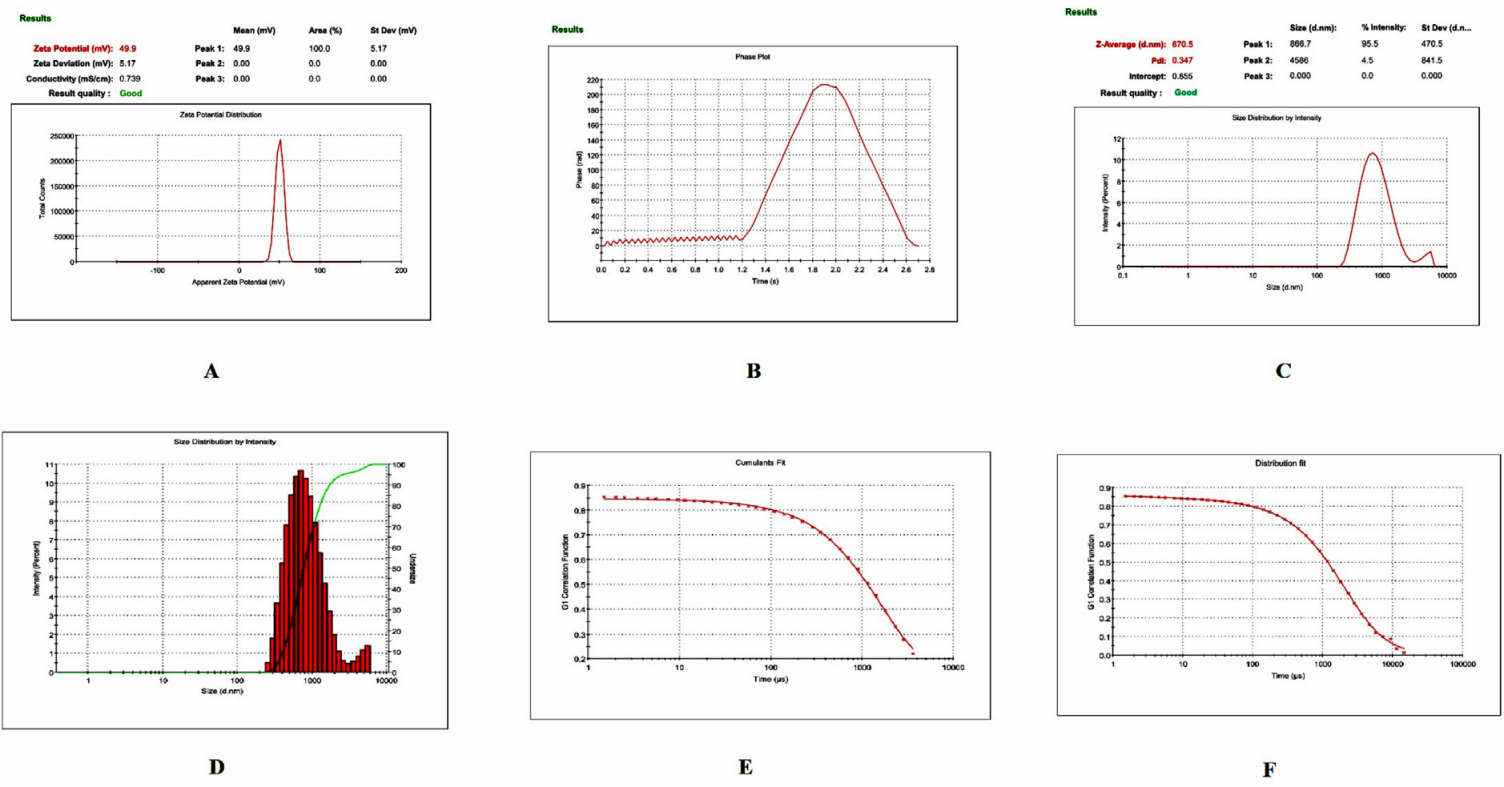
**Physical characterization of cisplatin-loaded PEGylated chitosan nanoparticles of batch 3** (A) Zetapotential analysis (B) Phase plot of particulate colloidal system (C) Particle Size distribution analysis through intensity (D) Particle Size distribution analysis (E) Cumulative fit of particulate colloidal system (F) Particle distribution fit of particulate colloidal system.

**Fig. 6:**
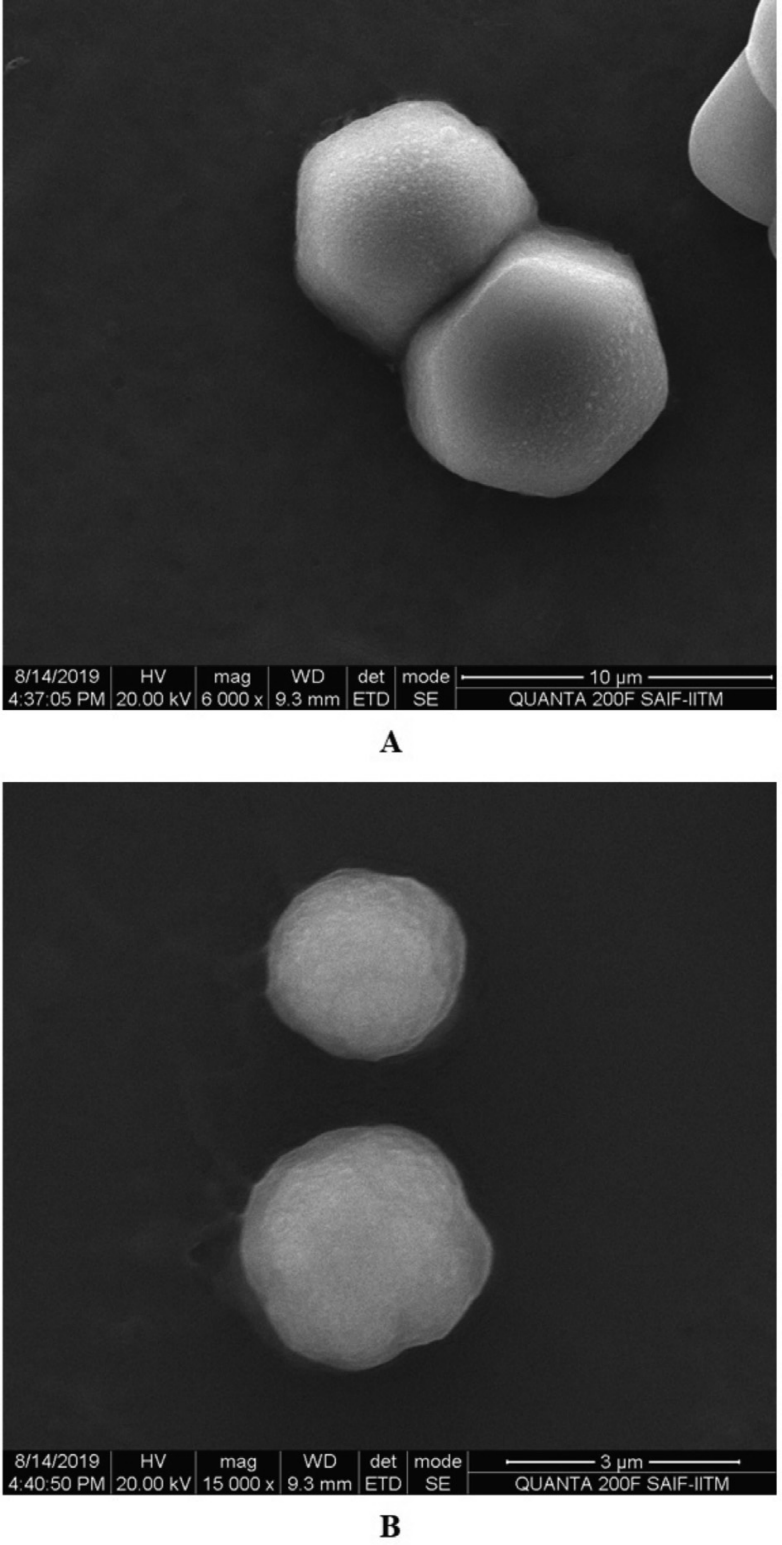
**Scanning electron micrograph study of batch 3** (A) The scanning electron micrograph of cisplatin-loaded PEGylated chitosan nanoparticles of batch 1 under 6, 000 × magnification (B) The scanning electron micro-graph of cisplatin-loaded PEGylated chitosan nanoparticles of batch 3 under 15,000 × magnification.

Batch 3 had a unique ZP of 49.9 ± 5.17 (Fig. 5A) and the uniform distribution of the particles is represented in phase plot of colloidal system (Fig. 5B). The nanoparticles were uniformly distributed with a PDI value of 0.482(Fig. 5C). Approximately 93.5% of these particles were 741.4 z. d.nm. The remaining 6.5% showed particle clumping. Zeta potential analysis confirmed the homogeneity of particles as it did not show any extra peaks Based on particle size and ZP analyses, batch 3 was observed to be the ideal batch to formulate cisplatin-loaded PEGylated chitosan nanoparticles. The Y-intercept value of the intensity peak is an important tool to estimate the signal:noise ratio of an instrument that measures the particle size intensity of samples and can be used to evaluate data quality. Generally, an ideal signal of a value greater than 0.9 is the best colloidal system. In this study, the nanoparticles prepared in all the batches exhibited Y-intercept values of 0.894, 0.893, and 0.855, respectively. In an earlier study, cisplatin-loaded chitosan nanoparticles using formaldehyde as a cross linker exhibited a similar Y-intercept value, of 0.920 (Abouelhag et al., 2017). Interestingly, the batch 3 particles were exhibiting smooth surfaces with spherical shape with minute pores (Fig. 6A) at 6000 × magnification. However, when the particles were observed under 15, 000 × magnification, the particles were smooth, spherical in shape and showing many pores on the surface. Although the physicochemical characterization of all three batches yielded positive results, batch 3 was deemed the optimal batch since the zetapotential analysis revealed that it was strongly cationic and had superior stability when compared to the other batches.

In this study, cisplatin-loaded PEGylated chitosan nanoparticles were formulated successfully at pH 6.5. Recently, a novel method was reported for the PEGylation of chitosan nanoparticles through ionic interaction and polymerisation (Bozuyuk et al., 2018). Their study demonstrated the preparation of PEGylated chitosan nanoparticles using methacrylamide. They showed that the formation of particle aggregation is influenced by the degree of methacrylation, pH, and free PEG on the chitosan nanoparticles (Bozuyuk et al., 2018). In contrast, this study developed different formulations based on the outcome of preformulation studies. Three formulations were designed based on chitosan: PEG: drug ratios. However, in these formulations, the cross-linker concentration was maintained at 0.25% v/v formaldehyde. The nanoparticles formulated in all three batches were successful at pH 6.5. However, batch 3 was the most successful formulation of nanoparticles, with a nano size particle and homogenous distribution. The ZP of batch 3 cisplatin-loaded PEGylated chitosan nanoparticles indicated that they were stable cationic particles. The cationic property of chitosan was not changed by the PEGylation of the chitosan polymer. The ZP is important for a successful targeted drug delivery system (Bozuyuk et al., 2018, Abouelhag et al., 2017). ZP is the degree of the electrostatic attraction between particles, which is the most important parameter for targeting at the cellular level because cell membranes are anionic (Shahed et al., 2017). Nanoparticles target and interact with the plasma membrane and penetrate the cell via endocytosis. The particles in all three batches exhibited unique ZPs between + 38 and + 49.9 mV. Nanoparticles with ZPs of greater than + 30 mV are strongly cationic and those with ZPs of less than −30 mV are strongly anionic (Jeffrey et al., 2011). This indicates that the nanoparticles developed in this study were stable formulations. A study reported the effect of PEG on chitosan for the formation of nanoparticles (Shahed et al., 2017). Their study demonstrated that the chitosan:PEG ratio affects the development of nanoparticles; however, they did not report the effects of the size and ZP of the nanoparticles. Our study demonstrated that a higher PEG concentration formed larger nanoparticles (Table 1). Nanoparticles prepared with a ratio of chitosan:PEG of 5:1 and 10:1 (batch 1 and batch 2, respectively) developed large discrete crystals. This was further demonstrated by SEM studies that showed crystals in both batches (Fig. 5, Fig. 6). However, in batch 3, the chitosan:PEG ratio was ideal as the particles exhibited a unique ZP and were nano sized. The particles were uniform and homogeneous, which was reflected in PDI, which is an important parameter for formulation and development, and SEM studies (Fig. 6). The morphological analysis of the particles showed a smooth, porous, and spherical shape with very little clumping (Fig. 6A). In Batch 3, most particles were nano size, homogenous, and spherical (Fig. 6B). Thus, batch 3 was the most ideal, even though the intensity peak showed that some particles were clumped. The batch 3 formulation passed the quality criteria of the instrument.

### 3.2 DSC analysis

**Fig. 7:**
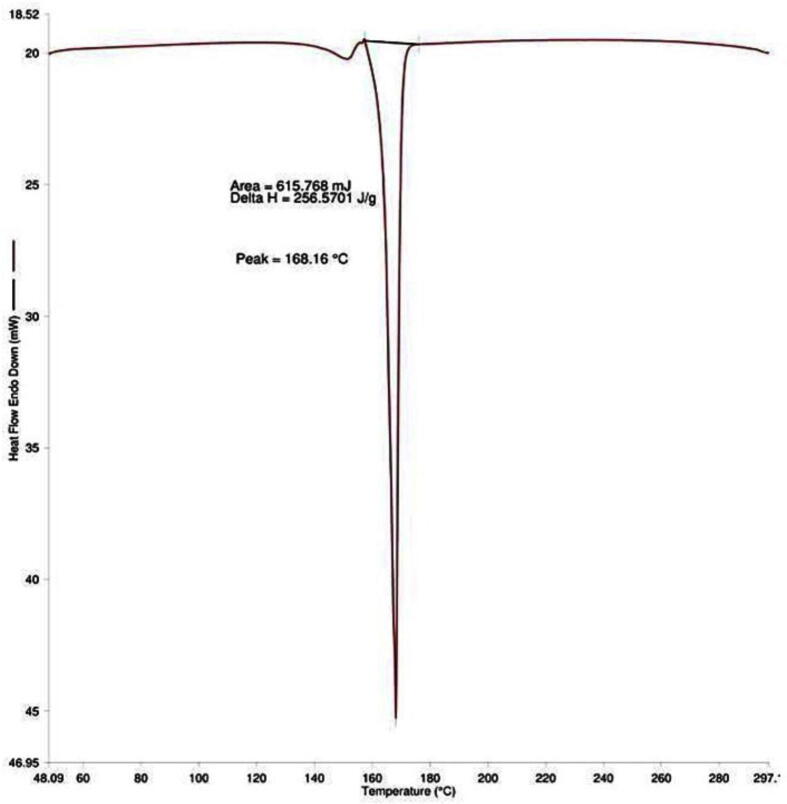
Differential scanning calorimetry analysis of cisplatin-loaded PEGylated chitosan nanoparticles of ideal batch.

The technique (DSC) estimates the changes in thermodynamic parameters like heat capacity, enthalpy, and entropy in nanoparticles because of changes in phase transitions, chemical reactions, and physical factors. The thermal degrading property of PEGylated nanoparticles was examined, and a molecular weight change was found, which resulted in the observation of a dramatic endothermic peak at 168.16 °C. However, the molecular weight change was initiated around 155 °C indicating the glass transition temperature which is represented as short peak in thermogram (Fig. 7). This demonstrates the thermal degradation property of the nanoparticles between 155 and 168.16 °C. The enthalpy of heat was calculated as delta H (area under the curve), which was 256.5701 J/g. In previous work, cisplatin-loaded chitosan nanoparticles exhibited an endothermic peak between 135.50 and 157.69 °C, confirming their thermal decomposition (Sultan et al., 2022).

An earlier study suggested that chitosan exhibits both an endothermic peak at the temperature of 152.20 °C and an exothermic peak at the temperature of 301.10 °C (Sobhani et al., 2017). According to the findings of research that was conducted by Yousefpour and colleagues and published in 2011, the thermogram obtained from differential scanning calorimetry showed that chitosan had an endothermic peak at 180 °C and an exothermic peak at 370 °C (Yousefpour et al., 2011). In contrast, the current study demonstrated that PEGylated chitosan nanoparticles were thermostable. This was shown by the fact that the endothermic peaks of these nanoparticles were observed at 168.16 °C, indicating that the PEGylation process modified the thermal stability of chitosan nanoparticles.

### 3.3 TGA analysis

**Fig. 8:**
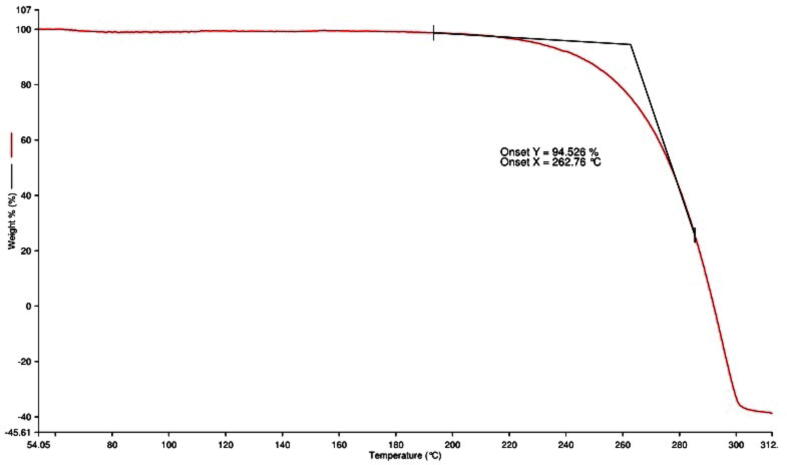
Thermo gravity analysis of cisplatin-loaded PEGylated chitosan nanoparticles of ideal batch.

TGA analysis was used to determine the thermal stability of the lyophilized PEGylated chitosan nanoparticles in the presence of oxygen, and the results showed a distinct peak consistent with a degrading effect (Fig. 8). Interestingly, the thermogram reveals that the weight loss of PEGylated chitosan nanoparticles was detected at a temperature of 262.76 °C, approximately 95% weight loss was observed demonstrating the thermal fragility of PEGylated chitosan nanoparticles at high temperatures. A previous study reported that the evaporation of water present in the chitosan polymer caused the initial breakdown of chitosan nanoparticles, which was began at 100 to 150 °C and resulted in a weight loss of 15%. The second stage took place at temperatures ranging from 150 to 650 °C and was characterized by the dehydration of the saccharide rings, depolymerization, and disintegration of the acetylated and deacetylated units. At 650 °C, there was still 30% polymer remaining (Hongcai et al., 2015). The current investigation shown that the PEGylated chitosan nanoparticles did not cause any initial degradation; nevertheless, 95% of the particles disintegrated at 262.76 °C, while 5% of the PEGylated nanoparticles were stable at that temperature. Therefore, the present study showed that the unique thermal degradation property indicated the influence of PEGylation on chitosan polymer. However, the temperature of 262.76 °C for degradation showed that the nanoparticles are highly stable as injectable nanoparticles.

### 3.4 XRD analysis

**Fig. 9:**
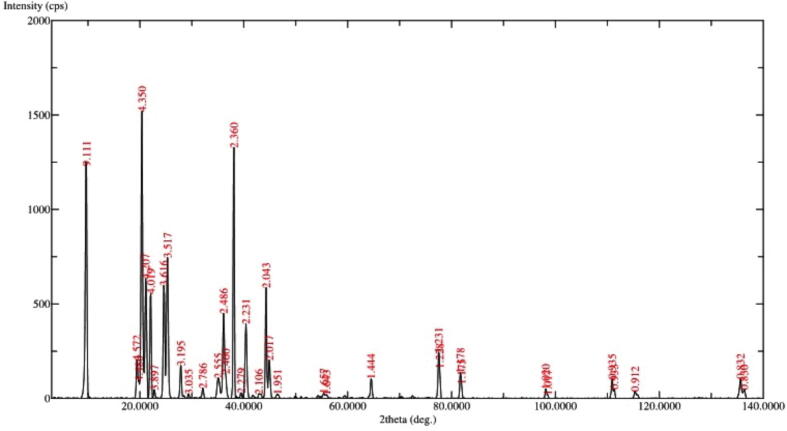
XRD analysis of cisplatin-loaded PEGylated chitosan nanoparticles of ideal batch.

The distinctive crystalline structures of nanoparticles can be characterized using XRD measurements. The XRD analysis performed at *2θ* revealed unique nanoparticles based on specific diffraction peaks in the current investigation (Fig. 9). The diffraction peaks (d-values) at 9.11, 4.350, 4.019, 3.517, 2.486, 2.380, 2.360, 2.043, and 1.44 ppm which confirmed the unique structure of PEGylated chitosan nanoparticles. The corresponding *2θ* values for d-values were 9.7°, 20.4°, 22.1°, 25.3°, 36.1°, 38.1°, 39.5°, 44.3°, and 64.5° respectively, while the relative intensity (I/Io) values of the corresponding d-values were 82, 100, 37, 50, 30, 88, 2, 39 and 7 respectively. Recently, the peaks at 35.38°, 37.47°, 49.29°, and 59.94° for cisplatin-loaded chitosan nanoparticles have been observed (Sultan et al., 2022). An earlier study suggested that the pure chitosan polymer displays two intense crystalline peaks at *2θ* value was 15.1° and 20.9° (Aziz et al., 2017). The PEG was exhibited at 9.318 °, 23.328 °, 26.408 °,36.288 °, 39.748 ° and 45.22 ° (Jayaramudu et al., 2016). An earlier study suggested that the *2θ* values of CS/TPP/DFO nanoparticles reached a maximum at 19.32°, 21.03°, 22.56°, 23.98°, and 28.40° (Lazaridou et al., 2020). In our study the sharp peaks were observed at 9.11, 4.350 and 2.360 indicating crystal particles, which was also observed in SEM studies. Recently we have reported the peaks at 35.38°, 37.47°, 49.29°, and 59.94° for cisplatin-loaded chitosan nanoparticles (Sultan et al., 2022). In the study by Maria Lazaridou et al., (2020) the *2θ* values of CS/TPP/DFO nanoparticles exhibited peaks at 19.32°, 21.03°, 22.56°, 23.98° and 28.40°. On the other hand, the CS/PEG NP exhibits prominent peaks at 19.10° and 23.25°. These peaks can be classified as chitosan fractions, while the lesser peaks at 26.10°, 30.85°, 36.15°, and 39.70° are indicative of PEG (Anand et al., 2018). In 2020 William et al., reported that the *2θ* values 8°-11° and 19°-21° of chitosan samples exhibited crystallite lattice. In the present study, the *2θ* values at 9.7°, 20.4°, 22.1°, 25.3°, 36.1°, 38.1°, 39.5° indicating the PEGylation of chitosan polymer. Thus, in the present study the *2θ* angle between 20.4°, 22.1°, 25.3°, 36.1°, 38.1° and 39.5° were showing the successful PEGylating of chitosan polymer and formed crystal particles with encapsulated cisplatin.

### 3.5 NMR studies

**Fig. 10:**
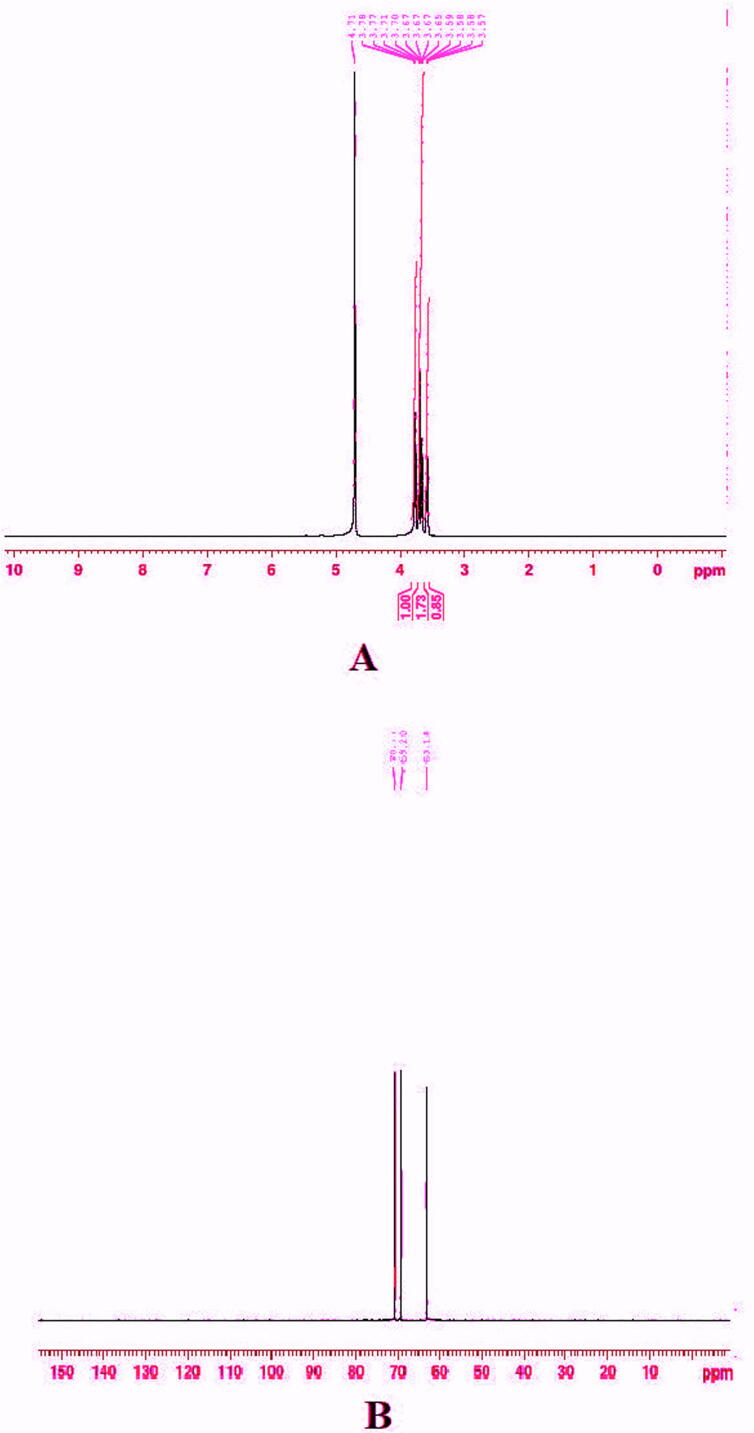
**NMR analysis**. (A) ^1^H NMR analysis of lyophilized cisplatin-loaded PEGylated chitosan nanoparticles of ideal batch (B) ^13^CNMR analysis of lyophilized cisplatin-loaded PEGylated chitosan nanoparticles of ideal batch.

The proton NMR (^1^H NMR) analysis gave the fingerprint region of PEGylated chitosan nanoparticles from 0.85, 1.73, and 1.00 ppm in the proton dimension. The most de-shielded proton peaks appeared at 3.57, 3.58, 3.58, 3.59, 3.65, 3,67, 3.70, 3.71, 3.77, 3.78 and 4.71 ppm (Fig. 10A). An earlier study suggested that ^1^H NMR spectrum showed peaks at 2.36, 3.52, 3.9, 4.2, 4.92 and 5.21 ppm during deacetylation processes (Lavertu et al., 2003). Later in 2006 Narayan et al., reported that chitosan polymer displayed peaks at 1.85, 2.95, 3.45, and 4.9 ppm. The shift in ^1^H NMR from 3.57 to 4.71 ppm indicated the presence of protons from the glycerol moiety of PEGylated chitosan nanoparticles. According to the findings of a previous investigation, a change in ppm concentration ranging from 5.10 to 3.70 indicated the existence of protons of the glycerol moiety (Alexandri et al., 2017). Another study reported that proton NMR of polyethylene glycol exhibited unique peak at 4.56 ppm (Julian et al., 1990). Furthermore, an earlier report suggested that PEGylated chitosan nanoparticles were showing characteristic peaks at 3.3 to 3.7 ppm, 4.3 to 4.5 ppm, 6.5–8.5 ppm (Meenakshi et al., 2013). The ^13^C NMR spectrum showed unique peaks that 63.18, 69.20, and 70.77 ppm (Fig. 10 B). An earlier study reported that ^13^C NMR of chitosan polymer showed various peaks at 67.7, 74.9, 81.4, 75.4, 59.6 and 102.7 ppm (Jing et al., 2016). The present study demonstrated that PEGylated chitosan nanoparticles were successfully formulated and not degraded after formulation The NMR study also confirming the crystal structure of nanoparticles.

### 3.6 FT-IR studies

**Fig. 11:**
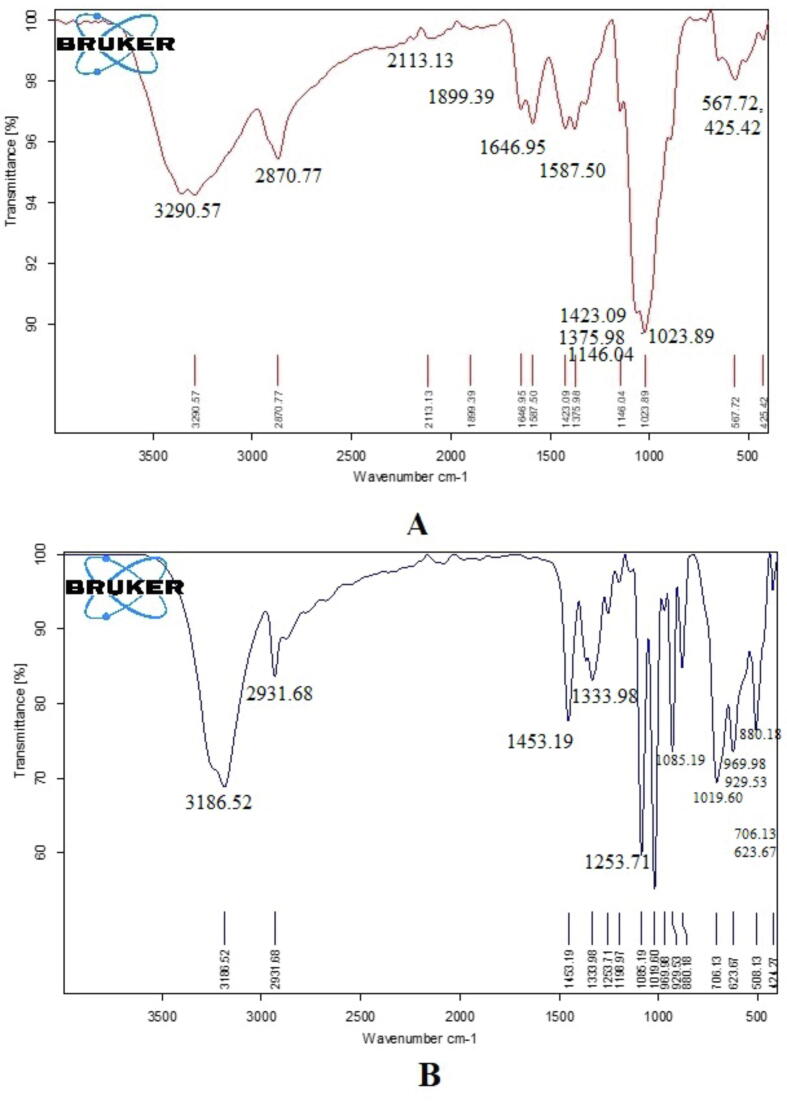
**FT-IR spectroscopy analyisis**. (A) FT-IR spectrum of Chitosan polymer (B) FT-IR cisplatin-loaded PEGylated chitosan nanoparticles.

The FT-IR spectrum of chitosan polymer and cisplatin loaded PEGylated chitosan nanoparticles are displayed in Fig. 11. The FT- IR spectra of chitosan polymer is showing characteristics bands at various frequencies (Fig. 11A). The large parabola shaped peak observed at 3290.57 cm^−1^ having stretching vibration indicating the presence of phenolic OH. The short peak at 2870.77 cm^−1^ indicates the presence of C–H stretching with an intramolecular bonding O–H stretching. The peaks at 1646.95 and 1587.50 cm^−1^ represents the presence of C = C stretching, conjugated alkene and N–H bending, amine respectively. The sharp fingerprint region at 1023.89 cm^−1^, C-N stretching depicting amine group. The studies that were conducted at frequencies of 567.72 and 425.42 cm^−1^ indicated several functional groups of organic compounds with an out-of-plane bending vibration. Rahul and Sugumar, (2020) reported the fingerprint regions of FT-IR spectroscopy of chitosan polymer. According to the findings of their research, Chitosan FTIR spectra exhibited sharp peaks at the following wavelengths: 564 cm^−1^ (out-of-plane bending NH, out-of-plane bending C–O), 711 cm^−1^ (out-of-plane bending NH), 1174 cm^−1^ (C–O–C stretching), 2865 cm^−1^ (CH2 stretching), and 3594 cm^−1^ (OH stretching). At 1604, 1598, and 1592 cm^−1^. Another study suggested that the FT-IR spectrum of chitosan polymer exhibited the finger print regions at 3441.01, 3259.70, 2927.94, 1662.64, 1554.63, 1404.18, 1033.86 and 867.97 cm^−1^ (Ahmed et al., 2016). In the current study the peaks of FTIR spectra demonstrated the characteristic features of chitosan polymer. The FT-IR spectra of cisplatin loaded PEGylated nanoparticles illustrates the presence of various fingerprint regions at 3186.52, 2931.68, 1453.19, 1333.98, 1253.71, 1085.19, 1019.60, 969.98, 929.53, 888.80, 706.13 and 623.67 cm^−1^. On comparing the FTIR frequencies of chitosan polymer and cisplatin loaded PEGylated chitosan polymer showed the shift from 3290.57 to 3186.52 cm^−1^, 2870.77 to 2931.68 cm^−1^, 1646.95 to 1453.19 cm^−1^, 1587.50 to 1333.98 and 1253.71 cm^−1^, 1023.89 to 1085.19 and 1019.60 cm^−1^, 567.72 and 425.42 to 888.80, 706.13 and 623.67 cm^−1^ indicates the cisplatin loaded PEGylated chitosan nanoparticles. In Fig. 11B the peaks appear at 1333.98, 1253.71, 1085.19, 1019.60, 969.98, 929.53 and 888.80 cm^−1^ demonstrating the PEGylation of chitosan. A recent report suggested that the PEGylation of chitosan, and this was confirmed by the emergence of distinctive peaks of PEG at 1386.56 cm^−1^, 1194.61 cm^−1^, 989.60 cm^−1^, and 874.06 cm^−1^ (Zaman et al., 2022). Furthermore, an earlier study suggested that 3990.18, 3400.19, 2878.01, 1657.01, 1382.29, 1082.22, and 577.47 cm^−1^ indicating the cisplatin (Othayoth et al., 2015). Therefore, the reports suggested that cisplatin loaded PEGylated nanoparticles were successfully formulated in this study.

### 3.7 *In vitro* release profile

**Fig. 12:**
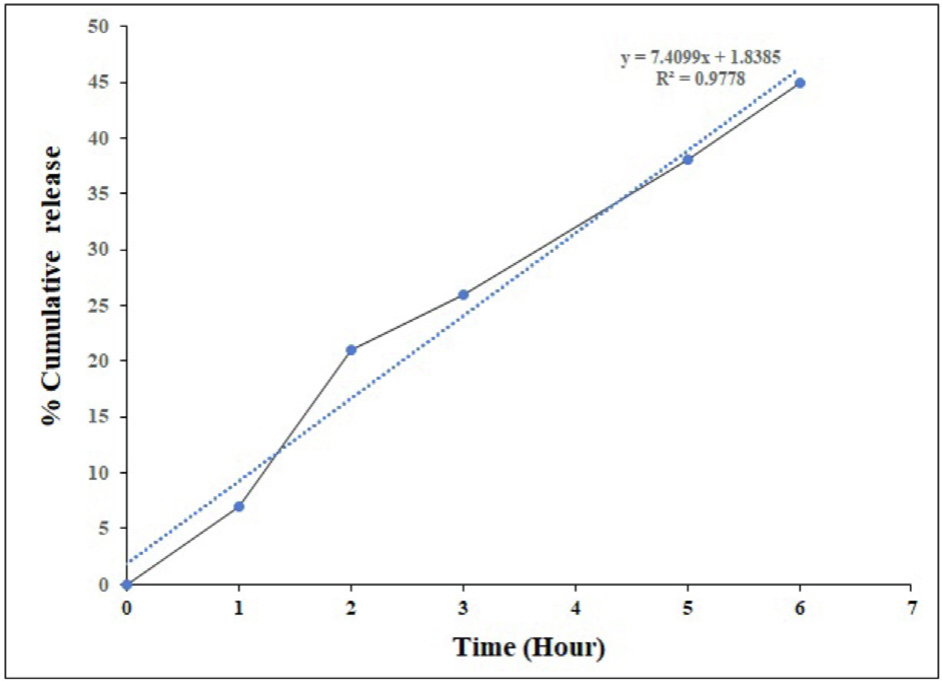
***In vitro* release profile**. Release kinetics of lyophilized cisplatin-loaded PEGylated chitosan nanoparticles of ideal batch.

Cisplatin was loaded successfully with an entrapment efficiency of 88.3 ± 2.5%, and 94.5 ± 1.2% loading was achieved. The *in vitro* release profile of PEGylated chitosan nanoparticles is presented in Fig. 12. The release of cisplatin from PEGylated nanoparticles was sustained manner but irregular fashion with an initial release of 4% in one hour. In our earlier study we achieved the entrapment efficiency of 83.3 ± 1.5%, and 92.0 ± 2.0% loading of cisplatin in chitosan nanoparticles. In the same study the percentage of drug lease from chitosan nanoparticles was observed as 7% in 30 min and sustained for 6 h (Sultan et al., 2022). In this study, the release profile followed linearity since the R^2^ value was 0.9778. Earlier study was exhibiting better linearity with an R^2^ value of 0.9322. However, the release pattern was showing initial burst release with initial in 30 min which was not observed in the present study. An earlier work shown that chitosan-TPP nanoparticles released 75% of their deferoxamine mesylate content in three hours (Lazaridou et al., 2020). Interestingly, in our previous study was able to release 43.8% of cisplatin from chitosan nanoparticles in 6 h. In contrast the present formulated PEGylated chitosan nanoparticles was able to release 48% in 6 h. The results demonstrate that cisplatin was released without any burst release from PEGylated chitosan nanoparticles and sustained to give a better release and this was reflected *in vitro* cytotoxicity study.

### 3.8 *In vitro* cytotoxicity

**Fig. 13:**
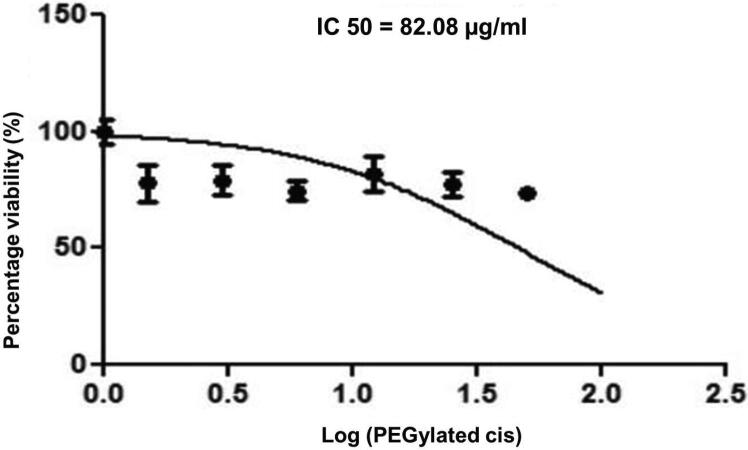
**Cytotoxicity study.** Dose-response curve of lyophilized cisplatin-loaded PEGylated chitosan nanoparticles of ideal batch against MCF-7 breast cancer cells.

*In vitro* cytotoxicity testing revealed that PEGylated chitosan nanoparticles inhibited the proliferation of MCF-7 ATCC human breast cancer cells. The IC_50_ value of value of PEGylated chitosan nanoparticles was determined at 82.08 ± 0.95 µg/mL, which shows cytoxicity effect (Fig. 13). However, in our previous study the IC_50_ value of cisplatin-loaded chitosan nanoparticles were highly significant with 4.085 ± 0.065 µg/mL. The concentration of cytotoxic effect was very high and showing less significant when compared to cisplatin-loaded chitosan nanoparticles (Sultan et al., 2022). The reason might be due to the high particle sizes with high cationic properties and therefore failed to show cytotoxic effect in low concentration. This can be attributed with the recent study which suggesting that the oxaliplatin individually exhibit the cytotoxicity had an IC_50_ value of 147.7 ± 63.91 µg/mL. However, the same study showed that after entrapping into chitosan nanoparticles IC_50_ value 23.88 ± 6.29 µg/mL against MCF-7 breast cancer cells (Fahmy et al., 2022). Their study suggested that the PEGylated chitosan nanoparticles showed better efficacy than non-PEGylated chitosan nanoparticles. The efficacy might be due to the penetration power of nanoparticles based on their characterization. Highly positively charged nanoparticles can easily target cells owing to the anionic nature of cell membranes. In the present study, the particles exhibited high cationic ZP, +49.9 mV. However, the release of drugs from nanoparticles is an important task that can be influenced by particle size (Gustafson et al., 2015, Sulin et al., 2015). In our study, the nanoparticles were too large to enter cells by passive diffusion. However, particles can also be internalized by active diffusion through electrostatic attraction between nanoparticles and cells. Therefore, batch 3 PEGylated chitosan nanoparticles may be able to successfully target cancer cells to deliver cisplatin at the cellular level.

## 4 Conclusion

Cisplatin-loaded PEGylated chitosan nanoparticles in an injectable dosage form can be successfully formulated using an ionic gelation technique. This study demonstrated that a low concentration of formaldehyde solution was able to link chitosan and PEG successfully. The nanoparticle colloidal injectable formulation was successfully prepared at pH 6.5 and 100% PEGylation was achieved without any loss of cisplatin. Physical characteristics, such as ZP, PDI, and particle size, which dictate the stability of nanoparticles, were favorable. Thus, the formulation will serve as a good therapeutic agent to deliver cisplatin safely. The analysis of cisplatin-loaded PEGylated chitosan nanoparticles' physicochemical properties demonstrated that these particles have substantial properties as injectable formulations. The cisplatin-loaded PEGylated chitosan nanoparticles exhibited lesser cytotoxicity on MCF-7 ATCC human breast cancer cells. Further studies can be established to assess the in vivo performance and pk parameters and cisplatin dose optimization. The results obtained in this study are promising to develop novel anticancer drug delivery system through PEGylation process of chitosan.

## Declaration of Competing Interest

The authors declare that they have no known competing financial interests or personal relationships that could have appeared to influence the work reported in this paper.
